# Biophysical modeling of anatomically realistic prenatal cortical folding development

**DOI:** 10.21203/rs.3.rs-8033969/v1

**Published:** 2026-01-12

**Authors:** Xianqiao Wang, Jixin Hou, Zhengwang Wu, Kun Jiang, Taotao Wu, Lu Zhang, Dajiang Zhu, Wei Gao, Mir Razavi, Tianming Liu, Ellen Kuhl, Gang Li

**Affiliations:** University of Georgia; University of Georgia; University of North Carolina at Chapel Hill; University of Georgia; University of Georgia; Indiana University - Indianapolis; University of Texas at Arlington; Binghamton University; University of Georgia; Stanford University; University of North Carolina at Chapel Hill

**Keywords:** cortical folding, growth heterogeneity, whole-brain computational model, symbolic regression, brain malformation

## Abstract

Cortical folds encode the architecture of human cognition, yet the mechanisms that transform the smooth fetal cortex into its convoluted geometry remain elusive. Biophysical modeling enables mechanistic insight into cortical morphogenesis, but existing models often lack anatomical realism and fail to capture key hallmarks and morphometrics of dynamic cortical folding in the developing human brain. Here, we introduce a novel whole-brain developmental framework that integrates region-specific, data-driven growth laws with anatomically accurate cortical geometry to enable realistic and biologically interpretable modeling of cortical morphogenesis during gestation. Growth fields derived from large-scale prenatal magnetic resonance imaging data capture spatiotemporal variations in cortical expansion and thickness across parcellated regions. Incorporating this heterogeneous growth yields anatomically faithful folding patterns that closely match qualitative landmarks and quantitative morphometrics from human imaging. Systematic perturbations of geometry and growth attributes delineate control parameters that produce realistic morphological variability and replicate clinically atypical brain phenotypes consistent with lissencephaly, pachygyria, and polymicrogyria. This framework provides a quantitative foundation for elucidating the mechanisms of typical and atypical fetal brain development and can serve as a promising generative engine for high-fidelity, longitudinal synthetic brain datasets to advance AI-driven developmental neuroscience and clinical translation.

## Introduction

The human brain undergoes remarkable morphological changes during gestation, giving rise to the highly convoluted folds of the cerebral cortex^1^. This folding process, which intensifies during the third trimester, coincides with rapid volumetric expansion and surface growth that foster neuronal proliferation and promote efficient information processing. Alongside the emergence of intricate folding morphology, cortical development exhibits pronounced spatiotemporal heterogeneity^2^. Cross-sectional neuroimaging analyses reveal distinct regional trajectories of cortical thickness and surface expansion across gestational ages^3,4^. This regionalization is further supported by transcriptomic and cellular evidence showing that spatially patterned gene expression regulates neural progenitors proliferate, differentiate, and form cortical layers, leading to divergent growth dynamics across cortical regions^5–7^ (Fig. S1). However, despite increasing evidence of heterogeneous growth signatures, how they physically translate into the complex folding patterns of the cortex remains poorly understood.

Cortical folding displays both consistent and variable features across individuals. Primary folds arise at stereotypical locations around mid-gestation, forming early landmarks such as the central sulcus and Sylvian fissure that are conserved across individuals. In contrast, secondary and tertiary folds emerge later, enriching local surface complexity and contributing to inter-individual variability^8,9^. This coexistence of regularity and variability points to a potential interaction between regional growth dynamics and geometric constraints^10^, yet their specific roles in shaping cortical folding patterns remain unclear. When normal developmental processes are disrupted, the resulting cortical malformations, characterized as atypical folding geometries, are clinically associated with neurodevelopmental disorders such as epilepsy, schizophrenia and autism spectrum disorder^11–13^. Clarifying the developmental mechanisms underlying these malformations is therefore crucial for uncovering their pathological origins and for informing early diagnosis and risk assessment.

With advances in computational and biophysical techniques, *in silico* modeling has emerged as powerful approach to simulating the complex spatiotemporal development of cortical folding within a physically grounded framework. To this end, various hypotheses and theories have been proposed to incorporate distinct biological mechanisms^10,14–18^. Among them, the prevailing explanation is the differential tangential growth hypothesis, which posits that deformation mismatch between the expanding cortical plate and the underlying white matter drives cortical gyrification^19–23^. While this paradigm has yielded important insight into folding mechanics, current models often yield simplified or generic patterns that fail to capture the anatomical complexity observed in human fetal magnetic resonance imaging (MRI) data. This discrepancy largely arises from the use of conceptual geometrical models and oversimplified growth representations. To approximate brain geometry, many models adopt generalized shapes such as spheres or ellipsoids^24–27^, which capture the brain gross morphology but lack regional anatomical details. Although some studies have employed reconstructed brain surfaces as simulation domains, they generally apply globally uniform growth assumptions derived from bulk volume approximations, which fail to account for region-specific developmental dynamics^10,22,23^. This raises a key question of whether uniting anatomically realistic brain geometries with biologically grounded growth representations can more faithfully capture cortical morphogenesis.

In this study, we present an advanced whole-brain computational modeling framework that integrates heterogeneous developmental growth laws to simulate the intricate prenatal cortical development with anatomically realistic folding patterns. Built upon prenatal brain geometries at 22 gestational weeks (GWs), the framework incorporates, for the first time, atlas-based regional growth laws derived from large-scale imaging-based measurements of cortical expansion and thickness spanning 22–40 GWs. We show that heterogeneous growth generates folding patterns with high anatomical fidelity, closely matching both cortical landmark features and quantitative morphological measures. Systematic variation of geometric factors, including the initial cortical surface configuration and cortical thickness, together with growth factors such as trajectory and magnitude, further demonstrates how their interplay shapes distinct folding morphologies. Beyond accurately modeling cortical development, our framework provides a generative platform for generating numerous anatomically realistic synthetic fetal brain datasets, overcoming the scarcity of longitudinal prenatal imaging. The framework also sheds insight into the mechanistic, evolutionary, and clinical aspects of brain morphogenesis while providing a scalable foundation for developing high-fidelity synthetic brain datasets that advance AI-based neuroscience research.

## Results

### Growth trajectories are heterogeneous across brain regions

**Figure 1:**
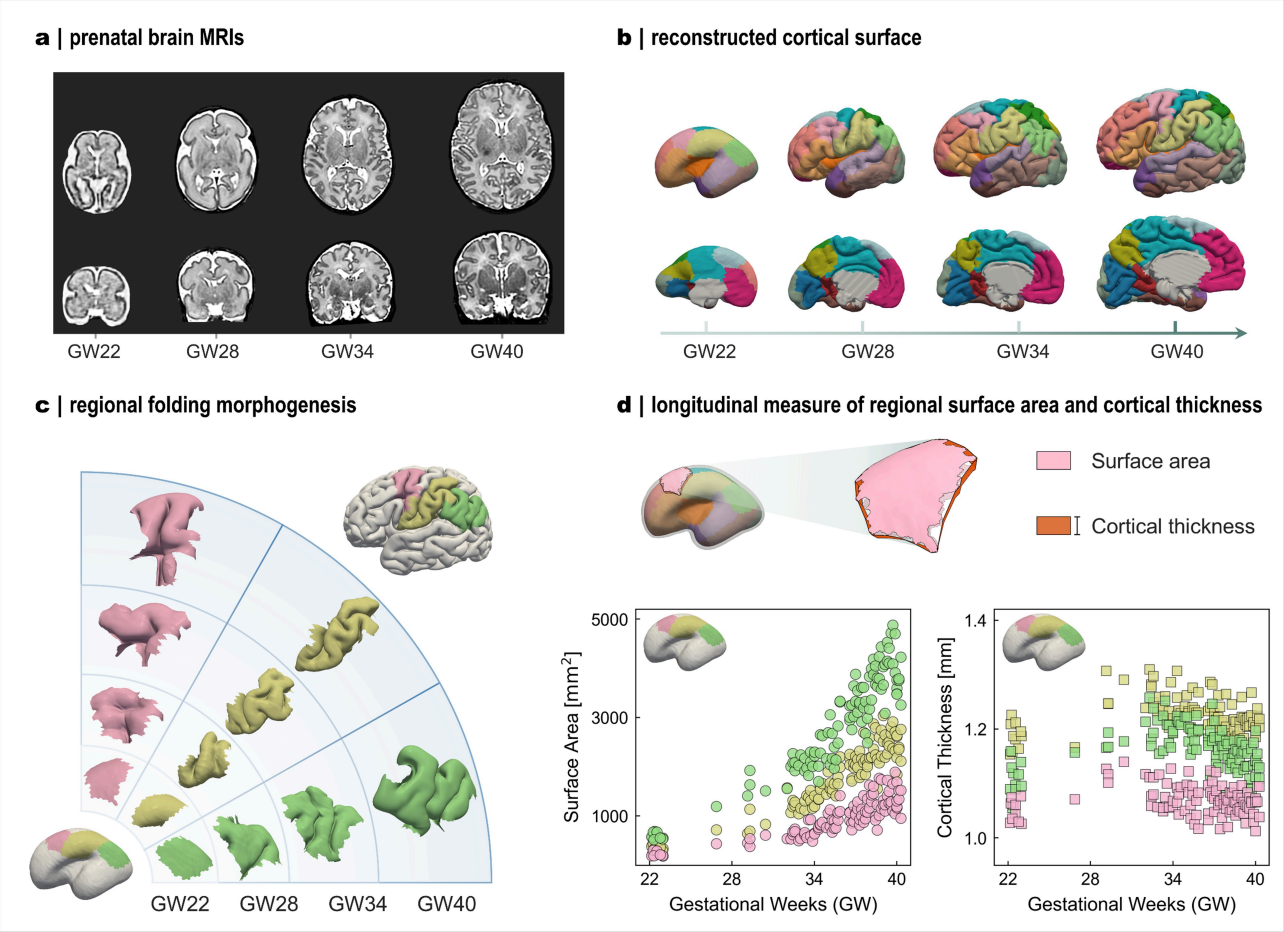
Heterogeneity of cortical development during the prenatal period. **a**: Prenatal MR images from 22 to 40 gestational weeks (GWs) in axial and coronal views. **b**: Reconstructed cortical surfaces from MRI data (two views), colors indicate regional parcellation. **c**:Regional morphogenesis of cortical folding during development, illustrated with three example regions. **d**: Developmental measurements of surface area and cortical thickness in the same three regions. Data were collected from 120 subjects.

**Figure 2:**
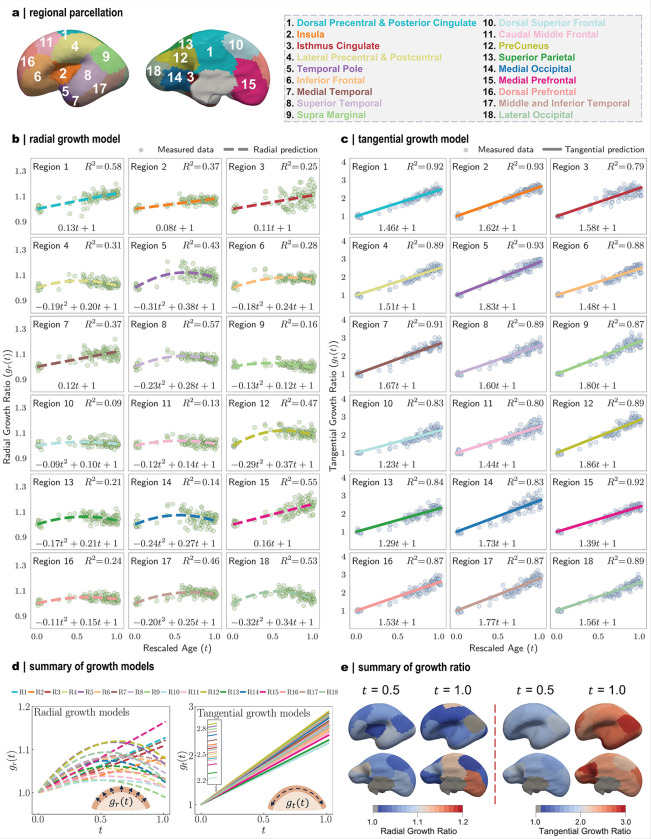
Characterization of region-specific growth models in the left hemisphere using symbolic regression. **a**: Developmental parcellation maps with corresponding anatomical regions. **b-c**: Growth models identified in radial and tangential directions. Dots denote MRI data from 22 to 40 GWs; lines indicate fitted growth models, with mathematic formulas shown at the bottom of each subfigure. *R*^2^ indicates fitting goodness. **d**: Summary of identified regional growth model in both directions. Insets illustrate schematic representations of radial and tangential growth, with the left inset showing a magnified view of the tangential growth ratio near t = 1. **e**: Growth ratio values visualized on the brain surface at 22 GWs for two developmental stages.

We first quantified regional cortical thickness and surface area in 120 fetal brains (22–40 GWs) curated from the developing Human Connectome Project (dHCP), using a developmental atlas specifically designed to parcellate the prenatal and neonatal cortex (Figs. 1a–d, 2a). Across 22–40 gestational weeks, these regions followed distinct morphogenetic trajectories, reflected by divergent patterns of cortical thickness and surface area (Figs. 1c–d). To facilitate cross-regional comparison, all measurements were normalized to their earliest-stage mean values (22 GWs), yielding relative growth ratios. Using a data-driven approach based on symbolic regression (Fig. S2), we derived mathematic models that capture regional growth dynamics, distinguishing radial growth (changes in cortical thickness; Fig. 2b) from tangential growth (changes in surface area; Fig. 2c).

Both radial and tangential growth exhibit marked heterogeneity in their developmental trajectories. Cortical thickness either increases steadily (e.g., region 1: dorsal precentral & posterior cingulate), reaches an early plateau (region 10: dorsal superior frontal), or follows a biphasic course. In the latter pattern, thickness peaks around mid-gestation (~ 31 GWs) and subsequently declines modestly (region 18: lateral occipital) or stabilizes at a plateau (region 17: middle and inferior temporal). These characteristic trends are consistent with previous fetal MRI observations^28–30^. The non-monotonic decline in thickness likely reflects localized microstructural remodeling processes, including synaptic pruning and dendritic compaction, that reduce apparent cortical thickness^31^. In contrast, tangential growth consistently follows linear trajectory, with rates substantially exceeding those of radial growth. This rapid tangential expansion during the prenatal period drives pronounced cortical enlargement, which in turn induces the emergence of folds. The resulting increase in cortical surface area accommodates proliferating neurons and establishes the structural foundation for nascent brain function^5^.

These regional distinctions demonstrate that cortical development varies across both space and time, resulting in spatiotemporal heterogeneity across brain regions (Fig. 2d). To further characterize these differences, we summarized the regional growth ratios at mid-gestation (31 GWs) and at term (40 GWs) based on the identified growth models (Fig. 2e). Tangential growth ratios are consistently higher than their radial counterparts, and their near-linear trajectories preserve the overall spatial distribution from mid-gestation to term. Notably, the temporal, parietal, and peri-Sylvian regions exhibit relatively higher tangential growth ratios. This observation aligns with neuroimaging evidence showing accelerated prenatal development in areas implicated in auditory and language functions^6,32,33^. Radial growth displays a distinct pattern: cortical thickness increases more rapidly around the central sulcus regions during late gestation, in line with the earlier maturation of primary sensorimotor cortices that support motor control and somatosensory processing^34^. Growth patterns are also not entirely symmetrical between hemispheres (Fig. S3). Although the exact trajectories differ, both hemispheres follow broadly similar trends, especially in tangential growth. Collectively, these findings reveal the pronounced spatiotemporal heterogeneity in cortical development, reflecting the inherent complexity underlying the emergence of human brain folding.

### Modeled folding patterns align with those observed in the developing brain

**Figure 3:**
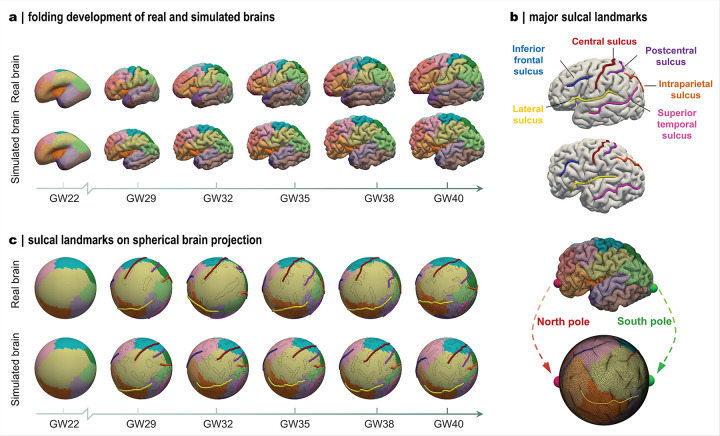
Modeled brains reproduce realistic cortical folding patterns observed in development. **a**: Longitudinal folding development of one simulated brain from 22 to 40 GWs and corresponding real brain folding patterns (from different individuals). **b**: Alignment of six sulcal landmarks in real and simulated brains. **c**: Longitudinal development of sulcal landmarks mapped onto the spherical projections of the brains shown in panel a. Major sulcal contours (black lines) were defined by regions with negative mean curvature. Location correspondence during spherical mapping is shown at right.

To examine how heterogeneous growth shapes cortical folding, we employed an *in-silico* modeling approach. A whole-brain computational framework was constructed with anatomically realistic geometry (Fig. S4), into which the identified regional growth models were incorporated as deformation drivers to recapitulate cortical folding development across mid-to-late gestation (22–40 GWs). Starting from an initial smooth geometry, the model progressively generated convoluted folds that conform to realistic brain morphology (Fig. 3).

The resulting brains closely resemble developing human brains not only in overall volumetric growth but, more importantly, in the emergence and evolution of major folding patterns across corresponding developmental stages (Fig. 3a). We note that reference brains shown here represent cross-sectional samples from different individuals and serve as developmental benchmarks rather than direct subject-level comparisons. The modeled trajectories indicate that primary folding patterns are largely established by ~ 32GWs, after which existing sulci mainly deepen and widen in concert with overall brain expansion. This developmental timeline aligns with *in utero* imaging observations showing that primary folds emerge by 32–34 GWs, while subsequent complexity arises through secondary and tertiary fold elaboration^35^. Notably, the framework reproduced key anatomical folding features, accurately capturing six major sulcal landmarks in both location and relative dimension (Fig. 3b). These sulci are well-recognized markers of cortical organization due to their structural and functional relevance. For example, the central sulcus demarcates the boundary between primary motor and somatosensory cortices, which are essential regions for motor control and sensory perception^36^, whereas the superior temporal sulcus represents a prominent landmark associated with higher-order auditory and language processing, as well as social cognition^37^.

To visualize and compare cortical folding patterns, we projected the brain morphologies onto a unit sphere with overlaid major sulcal contours (Fig. 3c). In the modeled brains, the relative positions of major folds stabilize by approximately 32 GWs; beyond this stage, the sulci primarily elongate and widen, while secondary and tertiary branches gradually emerge to further enrich folding complexity. By contrast, sulcal configurations in real brains appear less consistent across gestational ages, reflecting inter-individual variability inherent in cross-sectional datasets. Importantly, the modeling framework allows continuous tracking of folding evolution, generating brain morphologies at any developmental stage (Movie 1). This capability underscores a key strength of computational modeling in reconstructing the full longitudinal development of cortical folding from a single MRI scan.

**Figure 4:**
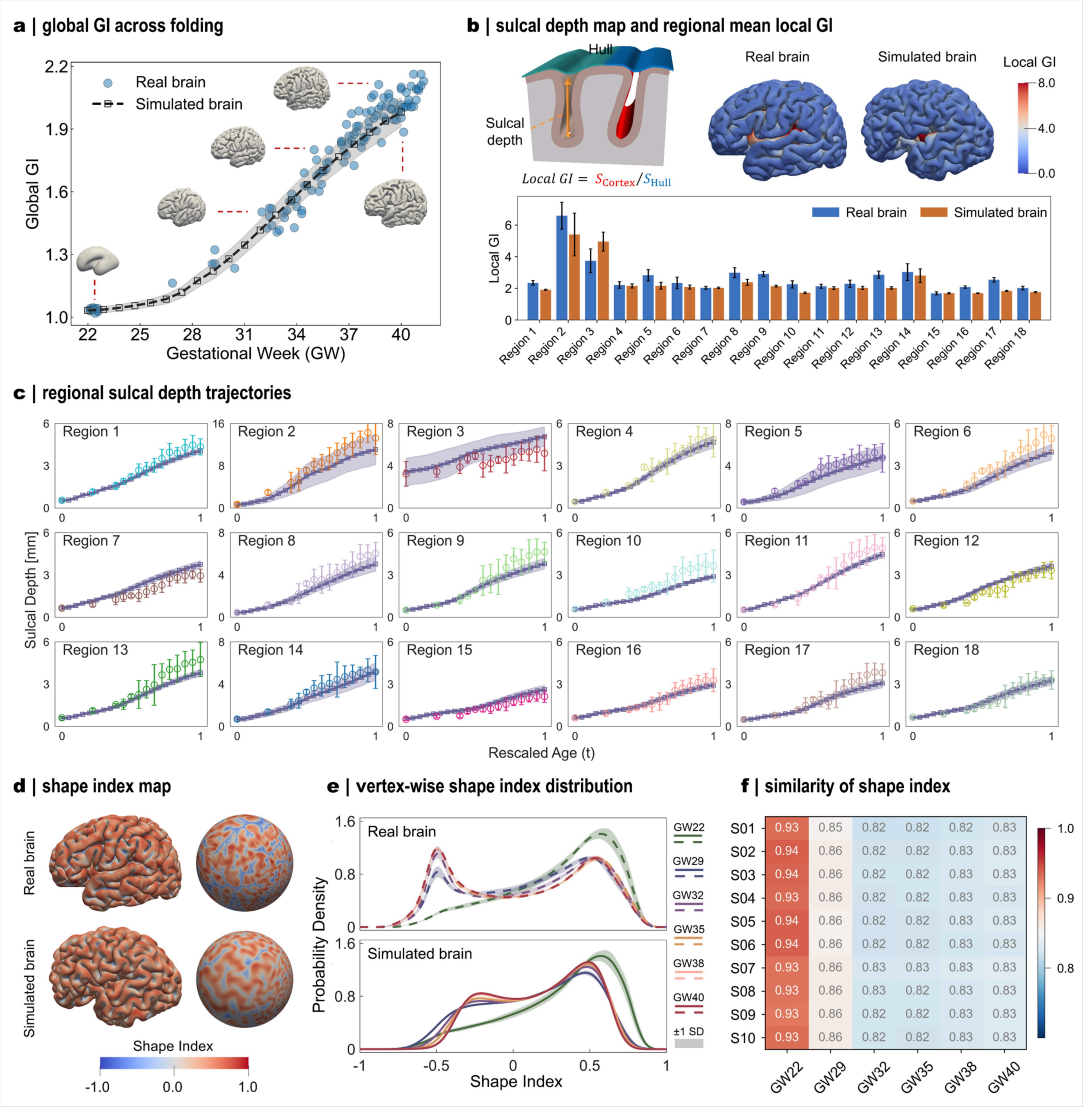
Modeled brains quantitatively recapitulate realistic folding patterns across scales. **a**: Developmental trajectory of global gyrification index (GI) in real and simulated brains. Insets show real brain folding patterns for typical data points. Simulation data are shown as mean ± s.d. (n=10). **b**: Schematic illustration of sulcal depth and local GI measurement (top left). Distribution of local GI in real and simulated brains at 40 GWs (top right) and summary of regional mean local GI across 18 regions (n=20; 10 subjects per case). **c**: Trajectories of regional mean sulcal depth across 18 regions. Simulation data are shown as mean ± s.d. (n=10). Dots and error bar indicate sulcal depth from 120 subjects. **d**: Distribution of shape index in real and simulated brains, displayed on folded and spherical surfaces. **e**: Shape index distribution at all vertices across six gestational ages with data are shown as mean ± s.d. **f**: Average similarity of shape index between ten simulated brains and real brains, corresponding to panel e.

To evaluate the accuracy of folding reproduction, we quantified multiple morphometrical parameters and compared results across global, regional, and vertex scales (Fig. 4). At the whole-brain level, folding complexity was assessed using the global gyrification index (GI), which measures the cortical surface convolution relative to the enclosing convex hull. During development, the global GI exhibits an accelerated near-linear increase after ~ 27 GWs, marking the onset of rapid fold formation (Fig. 3). Moreover, the global GI trajectories of the modeled brains closely mirror those of developing brains, reflecting consistent global folding dynamics across development (Fig. 4a). At the regional level, we evaluated the mean local GI and sulcal depth. The local GI refines the global metric by quantifying folding complexity within specific cortical territories, whereas sulcal depth directly measures the depth of individual folds (Fig. 4b). At term, local GI peaked prominently in the insular (region 2) and isthmus cingulate (region 3) areas, consistently with their persistent deep concavities throughout development. Overall, regional local GI values in modeled brains closely match those in developing brains, with relatively larger deviations in the peri-Sylvian and isthmus cingulate regions. Such discrepancies likely stem from geometric complexity that complicates both modeling and morphometric quantification. We further tracked regional sulcal depth trajectories across gestation for both modeled and real brains (Fig. 4c). Sulcal depth exhibited a biphasic growth pattern, characterized by an initial slow increase followed by accelerated deepening, with a transition occurring near 29 GWs (t ≈ 0.4). In most cortical regions, the modeled trajectories faithfully captured both the trend and magnitude of sulcal deepening observed in developing brains.

Subsequently, we evaluated folding accuracy at the vertex level using a curvature-based measure, the shape index. The shape index encodes local surface geometry, with positive values corresponding to gyri and negative values to sulci (Fig. 4d). In both modeled and developing brains, vertex-wise shape index distributions consistently show a characteristic double-peak profile centered around ± 0.5. This reflects the increasing prominence of ridge and rut features, consistent with previous reports^38,39^ (Fig. 4e). Marked shifts in the distribution are observed after the initial stage at 22 GWs, coinciding with the onset of surface buckling that initiates fold formation. As development proceeds, the distributions gradually converge in parallel with the maturation of major folds during late gestation. To quantify vertex-wise similarity, we computed a similarity index between each modeled brain and the average of ten real brains at the corresponding gestational stage (Fig. 4f). Across all stages, similarity indices exceed 0.8 for all brain subjects, indicating high vertex-level fidelity in the modeled brains. Together, these qualitative and quantitative comparisons demonstrate that the framework reliably generates anatomically realistic cortical folding patterns and serves as a powerful complement to longitudinal imaging datasets.

### Heterogeneous growth produces more realistic folding outcomes

**Figure 5:**
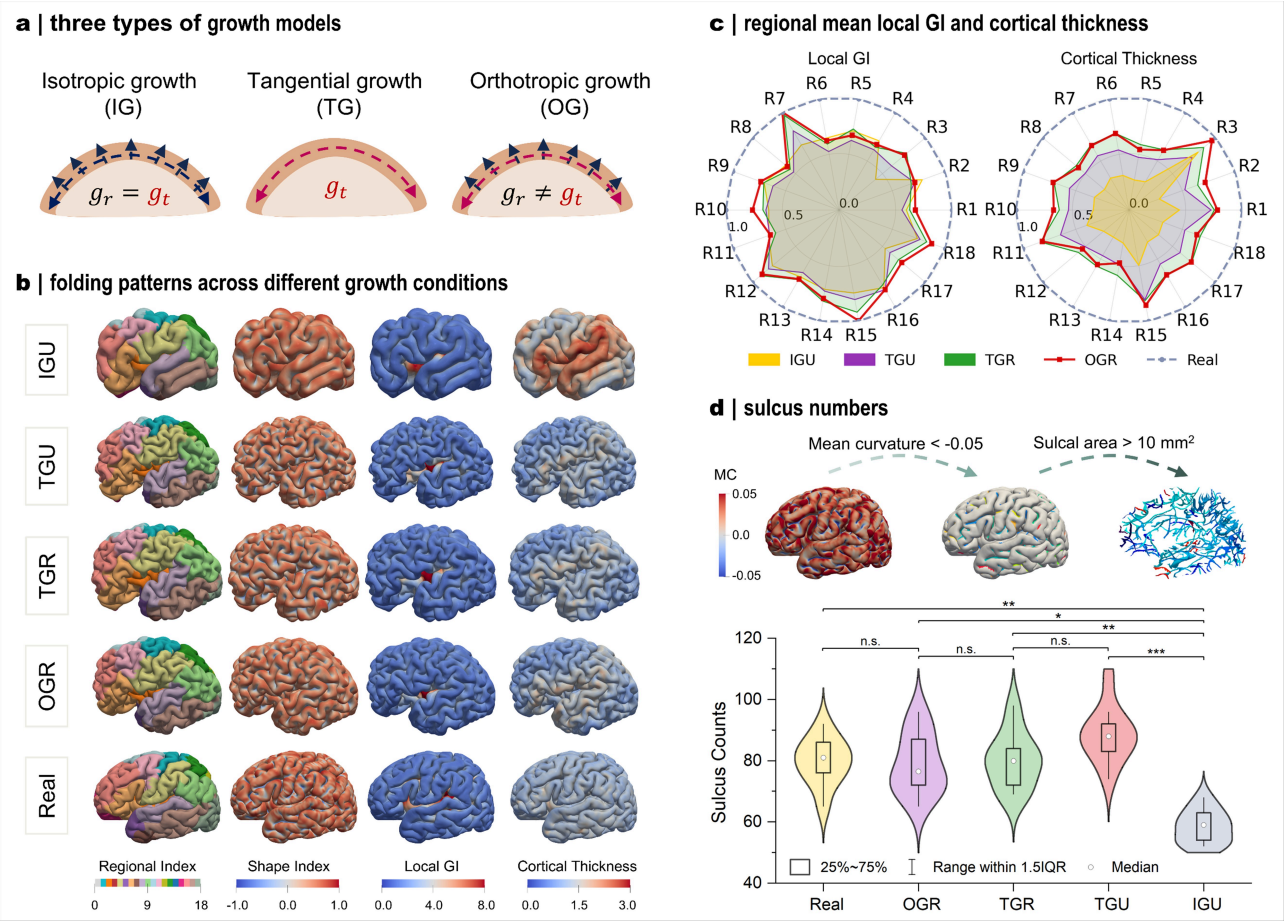
Heterogeneous tangential growth best captures realistic cortical folding patterns. **a**: Schematic illustration of three growth conditions. **b**: Simulated folding patterns: isotropic growth with uniform profile (IGU), tangential growth with uniform profile (TGU), tangential growth with regional profile (TGR), and Orthotropic growth with regional profile (OCR). **c**: Regional comparison of local GI (left) and cortical thickness (right). **d**: Sulci were defined as regions with mean curvature < −0.05 and area > 10 mm^2^. Sulcus numbers across the five cases in panel b are compared in violin plots (n = 50; 10 subjects per case). White dot indicates medians; black box represents interquartile range (IQR), covering the 25th to 75th percentiles; whiskers, 1.5 × IQR. Statistical significance was assessed by Kruskal–Wallis test with Dunn’s post hoc comparisons, with *P* values adjusted by Bonferroni correction. ***P < 0.001; **P < 0.01; *P < 0.05; n.s., not significant.

While the imaging-informed framework reproduces folding patterns with anatomical fidelity, the specific contribution of regional growth heterogeneity to this process remains to be systematically elucidated. To isolate this effect, we examined alternative growth modes by introducing isotropic (same in all directions) and tangential (restricted to tangential planes) formulation in addition to the orthotropic growth assumed in the cortical gray matter (Fig. 5a). Two growth profiles, uniform and regionally varying, were further applied across the cortical gray matter, yielding four modeling cases: isotropic growth with a uniform profile (IGU), tangential growth with uniform (TGU) or regional (TGR) profiles, and orthotropic growth with a regional profile (OGR). Among these, IGU and TGU represent the simplified growth assumptions commonly adopted in previous studies.

These growth conditions produce distinct folding outcomes (Fig. 5b). Under isotropic growth, the enhanced radial expansion yields less complex morphologies, characterized by bulky gyri and widely spaced sulci. Tangential and orthotropic growth generate broadly similar folding patterns, with TGR and OGR producing nearly indistinguishable morphologies. In contrast, TGR and TGU differ more noticeably, especially around the central sulcus, which was less developed in TGU. This observation suggests that regionalization of tangential growth contributes substantially to shaping cortical folding patterns. To quantitatively compare these effects, we analyzed regional averages of local GI and cortical thickness (Fig. 5c). Deviations under different growth conditions were assessed against real-brain metrics using a similarity score, where values closer to one indicate stronger correspondence. Among the tested conditions, OGR and TGR show the closest agreement with real-brain measures, consistent with their folding observations in Fig. 5b. Their close correspondence, despite differing only in the radial growth component, implies that thickness variation plays a relatively minor role in driving major fold formation^40^. By contrast, uniform growth conditions, particularly isotropic growth, exhibit poorer alignment across most regions, with pronounced discrepancies in cortical thickness. To further evaluate growth effects, we compared the number of major sulci in the left hemisphere, identified using empirical curvature and patch-area thresholds (Fig. 5d). As expected, IGU produces substantially fewer sulci than both real brain and other growth models, consistent with its sparse folding patterns. The remaining three conditions generated sulcus counts comparable to real brains, with TGU showing slightly elevated median values, though these differences were not statistically significant. Overall, these findings demonstrate that heterogeneous tangential growth is indispensable for reproducing realistic cortical folding development^19^.

### Anatomical geometry and regional growth shape cortical folding patterns

In this section, we investigate how anatomical configuration and growth models influence cortical folding outcomes. By systematically examining these factors, we demonstrate the framework’s capacity to generate diverse folding morphologies under varying control parameters and to reveal the developmental mechanisms underlying cortical folding.

**Figure 6:**
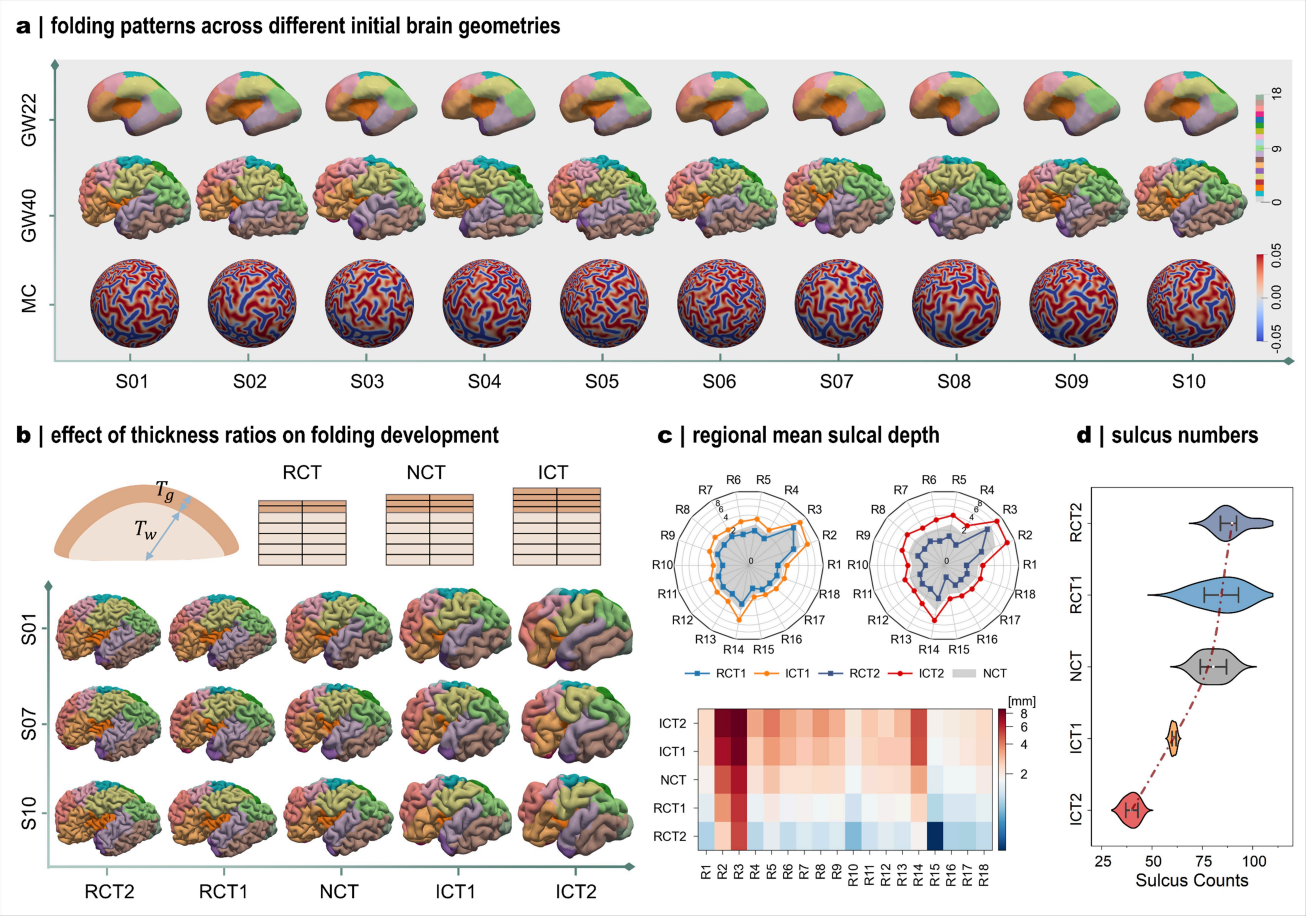
Anatomical geometry shapes cortical folding patterns. **a**: Influence of initial brain geometry on cortical folding development. Ten subjects with distinct initial geometry at 22 GWs and their corresponding folding patterns at 40 GWs are shown, with mean curvature (MC) distributions mapped onto spherical projections. Different initial geometries produced divergent folding outcomes. **b**: Influence of initial cortical thickness on folding development. Three schematic scenarios of varying cortical thickness are illustrated: reduced (RCT), normal (NCT), and increased cortical thickness (ICT). The modeled folding patterns from three representative subjects are shown below. For RCT1/ICT1 and RCT2/ICT2, the gray matter surface was offset inward or outward by 3 mm and 6 mm, respectively. Altering initial cortical thickness markedly modifies folding patterns. **c**: Comparison of the mean regional sulcal depth across the five thickness cases in panel b (n = 50; 10 subjects per case). **d**: Summary of sulcus numbers across the five cases, computed using the same method as in Fig. 5c. White dot indicates medians; black box represents interquartile range (IQR), covering the 25th to 75th percentiles; whiskers, 1.5 × IQR. The dash line indicates the increasing trend of sulcus numbers with decreasing initial cortical thickness.

We first examined the role of anatomical configuration, focusing on initial brain geometry and cortical thickness (Fig. 6). Owing to individual developmental variation, brain morphologies exhibit noticeable geometrical differences while remaining largely smooth at 22GWs (Fig. 6a). From a mechanical perspective, these variations translate into distinct initial geometries that influence mesh generation and introduce varying undulations capable of triggering different folding patterns. This effect is evident in the modeled folding outcomes across subjects (Fig. 6a). While localized folding patterns differ, major features such as primary sulci and gyri are consistently reproduced. This correspondence mirrors imaging observations and underscores the robustness of our framework in generating anatomically realistic brain morphologies.

We further assessed the influence of initial cortical thickness on folding patterns. Although this factor has been widely explored in theoretical analyses using simplified brain geometries such as spheres or ellipsoids^20,24,25^, it has not been systematically investigated in realistic brain models. To address this, we varied cortical layer thickness by offsetting the cortical surface inward or outward, thereby altering the initial cortical thickness (Fig. 6b). Two levels of offset were introduced: modest adjustments (ICT1, increased thickness; RCT1, reduced thickness) and larger adjustments (ICT2 and RCT2), corresponding to 40% and 80% of the mean initial cortical thickness. Increasing the relative thickness produces fewer folds, characterized by bulkier gyri and deeper sulci, whereas decreasing it generates more densely distributed folds with smaller gyri and shallower sulci. This observation aligns with core–shell buckling theory, which reveals that greater shell thickness leads to less complex folding patterns^41^. To quantify these effects, we measured regional mean sulcal depth and counted the number of major sulci in the left hemisphere. Sulcal depth increases systematically with initial cortical thickness across all regions, with larger relative thickness producing proportionally greater changes (Fig. 6c). Conversely, major sulci enrich as the relative thickness decreased (Fig. 6d), mirroring the observed morphological patterns. Together, these findings demonstrate that variations in either initial geometry or cortical thickness can generate distinct folding morphologies while preserving consistent major folding features observed in early developing brains.

**Figure 7:**
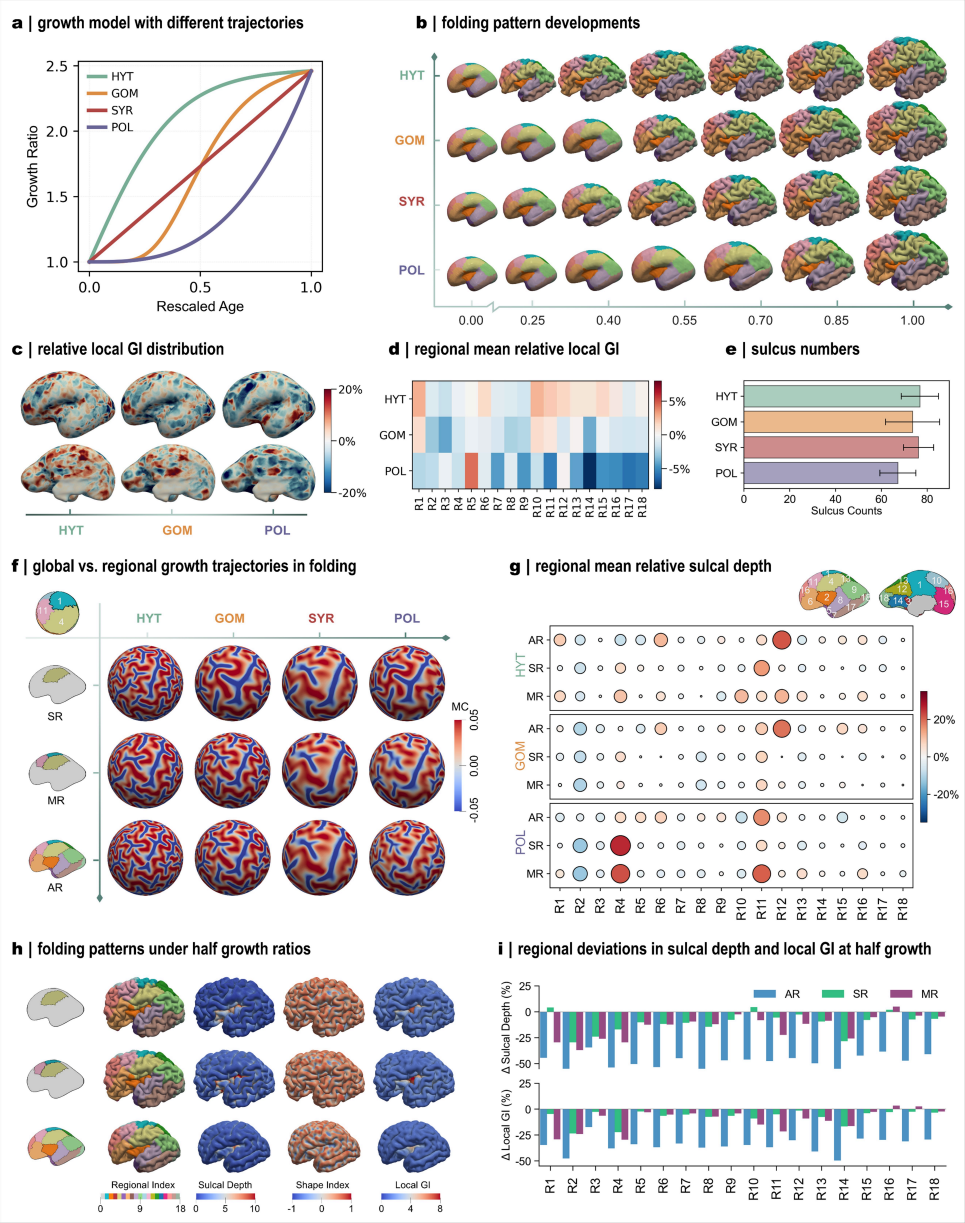
Regional growth model modulates cortical morphology. **a**: Growth models with identical final ratios but different trajectories: hyper tangent (HYT), Gompertz (GOM), symbolic regression characterization (SYR), and polynomial (POL). Growth model of region 4 is shown as an example. Trajectory variation was applied only to tangential growth, while radial growth remains fixed. **b**: Simulated folding development for different growth trajectory scenarios, with trajectory variation applied to all cortical regions. **c**:Relative local GI distributions at the final stage. Folding results of SYR serve as reference; all data are projected onto the initial brain geometry. **d**: Average relative local GI across regions. **e**: Sulcus numbers across growth models, using the same method as Fig. 5c (n = 20; 5 subjects per case). **f**: Regional vs global variation of growth trajectories. Cartoon illustrates perturbed trajectories for single region (SR), multiple adjacent regions (MR), and all cortical regions (AR). For regional variation, only selected regions deviated while others followed SYR. Folding patterns are shown with mean curvature (MC) mapped onto spherical projections. SYR results were repeated three times for comparison. **g**: Average relative sulcal depth across regions for different trajectory and regional variation cases. Circle size indicates magnitude of deviation (absolute relative error) from SYR, and color indicates direction of difference. **h**: Regional versus global variation of growth ratios. Folding patterns and metric distributions are shown for the same three scenarios as in panel f. **i**: Deviation of regional mean sulcal depth and local GI from SYR reference.

Next, we investigated the role of growth models in directing regional deformation during development (Fig. 7). This analysis is pertinent to developmental delays observed in neurodevelopmental disorders such as autism spectrum disorder^11^ and schizophrenia^12^, both linked to atypical cortical growth trajectories during prenatal stages. Unlike the growth conditions examined in the previous section, here we focused specifically on growth functions, considering two attributes: growth trajectories and growth values. Because thickness variation plays only a minor role in shaping major folding patterns, all modifications were applied exclusively to tangential growth models.

To examine the influence of growth trajectories, we introduced three representative temporal profiles, namely the hyperbolic tangent (HYT), Gompertz distribution (GOM), and polynomials (POL) distributions, while maintaining identical final growth ratios (Fig. 7a). These trajectory alterations were applied globally across all cortical regions. Altering the growth trajectories produces noticeable changes in the timing of fold emergence (Fig. 7b): the onset of folding varies among cases, following the temporal characteristics of each trajectory, yet the final folding morphologies remain largely similar (Movie 2). To quantify local differences, we calculated relative percentage errors in local GI with respect to the baseline trajectory (SYR) (Figs. 7c–d). Substantial deviations were observed for HYT and POL in both vertex-wise and regional comparisons, with HYT generally overestimating and POL underestimating local GI values, consistent with their growth divergence from the baseline growth trajectory. In contrast, the number of major sulci was comparable across all cases, though POL tends to yield slightly fewer sulci (Fig. 7e). These findings suggest that growth trajectories primarily modulate localized folding characteristics while exerting relatively minor influence on global folding organization^25^.

Because the trajectory alterations above were applied globally, we next examined the effects of regional perturbations on folding evolution. Two cases were considered: alteration within a single region (SR) and alteration across multiple adjacent regions (MR), with all other regions following the baseline trajectory. Folding morphologies were projected onto a spherical surface to visualize sulcal regions, defined by negative mean curvature (Fig. 7f). Regional alterations produce distinct sulcal patterns compared to the global alteration case (AR), particularly under single-region modification. Quantitative analyses reveal that trajectory alterations influenced not only the perturbed regions, but also their immediate neighbors, while distant regions (e.g., R14: medial occipital, R17: middle and inferior temporal, and R18: lateral occipital) show minimal changes in sulcal depth (Fig. 7g).

We then explored the effect of growth ratio magnitude on folding outcomes. Reducing the growth ratios by half, either regionally or globally, results in less developed folds within the affected regions, whereas unaffected areas remain largely unchanged (Fig. 7h). Relative deviations again reveal that regional alterations also propagate to neighboring areas (Fig. 7i). In summary, these analyses indicate that growth ratio magnitude predominantly governs the formation of major folding patterns, while growth trajectories modulate localized folding features, as reflected by local quantitative deviations. On this basis, we investigated hemispherical symmetry in folding patterns, as the two hemispheres can differ in both geometry and growth profiles even for the same subject^42^(Fig. S5a). The modeled brain showed asymmetric folding patterns, while preserving consistent major features across hemispheres (Fig. S5b). Although measurable differences were detected in both folding morphology (Fig. S5c) and regional measures (Fig. S5d), these did not reach statistical significance (Fig. S5e).

### Our modeling framework well captures abnormal cortical folding in malformations

**Figure 8:**
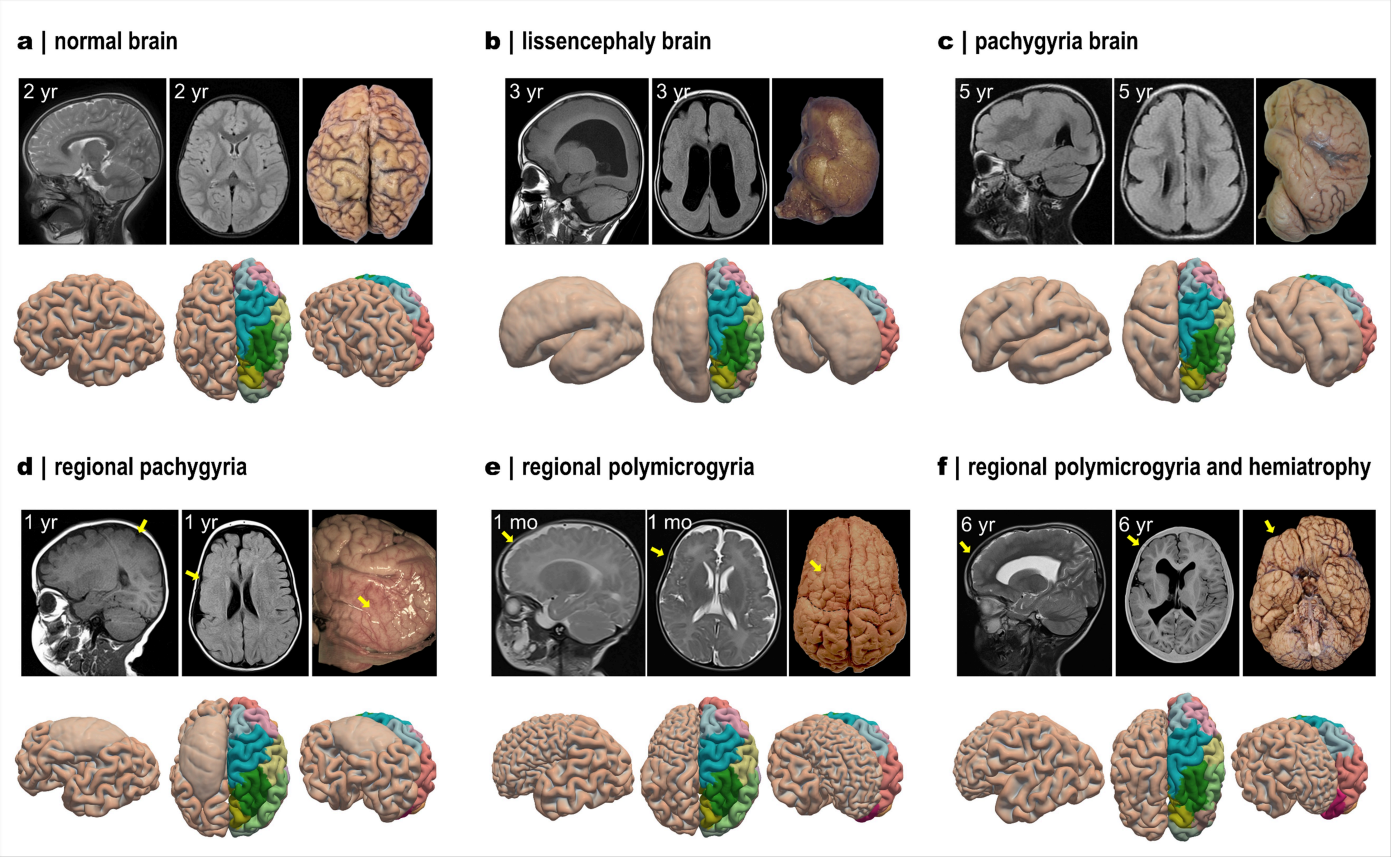
Modeling framework captures abnormal folding patterns in congenital brain malformations. Representative radiological and gross anatomical views of malformation cases are shown in panels a–f (top), alongside corresponding simulations generated with our whole-brain model (bottom). In each case, sagittal and axial MRI scans are provided together with gross anatomical photographs. **a**: Normal developing brain at 2 years of age (MRI case courtesy of Dalia Ibrahim, Radiopaedia.org, rID: 212958; anatomy image reproduced from Ref.^45^). **b**: Extreme lissencephaly brain at 3 years (MRI case courtesy of Ammar Haouimi, Radiopaedia.org, rID: 194766; anatomy image reproduced from Ref.^46^). **c**: Less severe lissencephaly (pachygyria) brain at 5 years (MRI case courtesy of Senai Goitom Sereke, Radiopaedia.org, rID: 181664; anatomy image reproduced from Ref.^47^) **d**: Regional pachygyria at 1 year (MRI case courtesy of Ammar Haouimi, Radiopaedia.org, rID: 67632; anatomy image reproduced from Ref.^48^). **e**: Regional polymicrogyria at 1 month (MRI case courtesy of Huda B. Gharbia, Radiopaedia.org, rID: 88483; anatomy image reproduced from Ref.^49^). **f**: Regional polymicrogyria with concomitant right hemispheric atrophy at 6 years (MRI case courtesy of Fazel Rahman Faizi, Radiopaedia.org, rID: 67168; anatomy image courtesy of Frank Gaillard, Radiopaedia.org, rID: 27813). In the simulations, brains were rendered in three orientations with region-based coloring; all pathological regions are located in the right hemisphere, which was horizontally flipped to facilitate direct comparison with the anatomical views. Yellow arrows highlight the regional malformations, corresponding to the case descriptions provided in Supplementary Table S1.

Through the preceding analyses, we demonstrated that the framework can generate diverse brain morphologies with realistic folding features by varying geometrical factors and regional growth models. Building on this, we further show that by adjusting these control parameters according to expert case descriptions derived from MRI scans (Table S1), the framework can capture abnormal folding phenotypes of typical malformations (Fig. 8). Representative examples include lissencephaly^43^ and polymicrogyria^44^, which are characterized by atypically reduced or excessive cortical folds compared to normal brain morphology shown in Fig. 8a. For example, Fig. 8b illustrates a severe lissencephaly case presenting a smooth cortical surface and markedly increased initial cortical thickness. This phenotype was reproduced in our framework by doubling initial cortical thickness and reducing regional growth ratios to 30% of their normal values (Movie 3). Similarly, pachygyria, a subtype of lissencephaly in which major sulcal landmarks are preserved despite an overall smoother surface, was recapitulated by applying the same two-fold increase in initial cortical thickness while maintaining normal regional growth models (Fig. 8c) (Movie 4).

Beyond global malformations, our framework can also capture region-specific developmental deficiencies. In one case, pachygyria is localized to the frontal and parietal lobes of the right hemisphere and presented as a smooth cortical surface with preserved thickness (Fig. 8d). To replicate this phenotype, we exclusively reduced the tangential growth ratios by half in the affected regions (R1: dorsal precentral & posterior cingulate, R4: lateral precentral & postcentral, and R11: caudal middle frontal) (Movie 5). Conversely, the regional polymicrogyria case, which exhibits abnormally dense but shallow folds in the right frontal lobe (Fig. 8e), was recreated by reducing initial cortical thickness by 40% in selected frontal regions while keeping all other parameters unchanged (Movie 6). Another polymicrogyria case, accompanied by volume atrophy in the right prefrontal cortex (yellow arrow in Fig. 8f), was reproduced by reducing initial cortical thickness by 40% in regions R6 (inferior frontal) and R16 (dorsal prefrontal) and halving both cortical and subcortical growth ratios (Movie 7). Although these reproductions remain qualitative owing to the limited availability of MRI data, particularly longitudinal scans, the results underscore the framework’s capability to replicate malformation-specific folding phenotypes. By linking growth dynamics and anatomical configuration to pathological morphology, this approach provides a mechanistic basis for interpreting cortical malformations within a unified developmental framework.

## Discussion

Cortical folds encode critical information about brain structural organization and functional specialization, yet their formation mechanisms remain poorly understood. While multiple factors such as neuronal migration, axonal tension, and cranial constraints have been implicated in shaping folding, mechanical forces arising from differential growth between gray and white matters are increasingly recognized as key drivers of cortical morphogenesis^50^. Previous computational efforts have provided valuable insight into this process but often lacked anatomical fidelity and fell short of reproducing the spectrum of folding patterns observed in the developing human brain^21,27^. In this study, we introduced a whole-brain computational modeling framework that integrates heterogeneous, region-specific growth models with anatomically realistic prenatal brain geometries, thereby unprecedentedly enabling faithful simulation of developmental folding trajectories documented in MRI datasets. The framework captures the spatiotemporal complexity of cortical morphogenesis, achieving anatomical fidelity in both the qualitative folding features and the quantitative morphometric measures.

Our findings demonstrate that tangential growth is the dominant driver of cortical folding, in agreement with both neuroimaging evidence and theoretical predictions^51^. Importantly, the inclusion of regional heterogeneity in tangential growth is essential for reproducing realistic folding morphologies. In the developing human brain, such heterogeneity is well documented and primarily arises from regionalized gene expression^52^ and molecular specialization^53^ across cortical areas. For example, spatial transcriptomic profiling has revealed that the prenatal cortex exhibits area- and layer-specific gene expression programs as early as mid-gestation, with frontal enrichment of genes such as CBLN2 and CYP26A1 and sharp transcriptional boundaries emerging between adjacent visual areas^7^. These spatially patterned molecular programs give rise to developmental heterogeneity across cortical regions, reflected in tissue-level organization such as axonal connectivity and vascular architecture, which together shape diverse folding morphologies^54^. Despite its biological relevance, growth heterogeneity has been underexplored in computational modeling of cortical folding. This lack of focus largely stems from the scarcity of high-quality MRI data and the inherent challenges of incorporating growth heterogeneity into anatomically realistic simulations^22^. Previous studies often relied on highly simplified geometries or imposed periodic growth fields on planar or patch-based models, limiting their ability to capture the complexity of cortical morphogenesis^25,55^. Such oversimplifications, while mathematically tractable, yield insights that are difficult to generalize to realistic developmental scenarios. Our framework directly addresses these limitations. By constructing anatomically faithful whole-brain models and MRI-based regional parcellations, we are able to assign growth laws derived directly from actual developmental trajectories into biophysical simulations. This approach provides, for the first time, a means to mechanistically examine how heterogeneous growth shapes cortical folding within a realistic developing brain. Notably, the framework offers a flexible platform that can be extended to incorporate other region-specific properties, such as spatial variations in material or cellular composition, provided reliable anatomical measurements are available. Beyond such extensions, it also enables mechanistic exploration of how intrinsic tissue properties, local growth dynamics, and emergent geometric constraints interact during cortical morphogenesis.

Both anatomical configuration and growth models critically shape cortical folding patterns. By systematically varying these factors, we showed that the framework can generate diverse yet realistic folding morphologies. This capability not only advances our understanding of the fundamental determinants of folding development but also, more importantly, provides a reliable platform for generating large-scale, high-fidelity synthetic brains with continuous developmental trajectories. Such synthetic datasets could serve supportive educational purposes, for instance by providing intuitive visualizations of cortical morphogenesis and helping learners or practitioners better understand developmental concepts. Moreover, they can effectively supplement the limited availability of longitudinal brain imaging datasets by supplying anatomically faithful inputs for ML models that predict cortical folding development, a particularly valuable contribution given the computational cost of biophysical simulations. Recent advances in ML models, including GAN-based generative frameworks^56,57^, physics-informed neural networks (PINNs)^58^, transfer-learning approaches^59^, and sequence-learning architectures such as PointNet–LSTM^60^, have shown strong promise for capturing and forecasting cortical morphogenesis across gestation. Our framework provides biologically grounded synthetic data to train and validate these approaches. When extended to simulate patient-specific or atypical developmental patterns, it could generate abnormal developmental datasets to train predictive models for early detection and intervention in neurodevelopmental disorders.

Beyond the forward process of predicting folding patterns from initial brain morphologies, our modeling framework also suggests the potential for reverse inference, thus estimating early brain states from mature cortical configurations. Because the simulations are constrained by biologically informed growth dynamics and biophysical principles, they preserve surface topology and generate smooth geometric transitions, thereby maintaining a coherent developmental trajectory from fetal to adult stages. This continuity mirrors realistic longitudinal development and opens the possibility of reconstructing early anatomy from later morphologies. This strategy resonates with the broader concept of “reverse engineering the brain”^61^, here applied to cortical morphogenesis, where mature folding patterns are used to infer plausible early anatomical states. Although still speculative, this direction could yield extensive physically grounded representations of fetal brain morphology. Such synthetic datasets can alleviate the scarcity of fetal imaging and provide valuable context for studies of neonatal development and adult brain disorders rooted in early developmental deficits. Furthermore, this approach also holds promise for advancing evolutionary neuroscience, for example by informing the interpretation of fossil endocasts^62^ and the reconstruction of extinct or ancestral brain states^63^.

Our framework can qualitatively replicate abnormal folding phenotypes, including lissencephaly, pachygyria, and polymicrogyria, by modifying growth laws or geometric parameters according to clinical case descriptions. These reproductions imply how malformation-specific perturbations give rise to distinct phenotypes. Clinically, such models could support early risk stratification and diagnosis by evaluating malformation progression from prenatal scans, offering a complementary tool for obstetric imaging and counseling. Beyond gross shape changes, certain conditions such as autism are also associated with localized variations in folding patterns^64^. While our framework does not directly capture such subtle cases, it provides a potential mechanical interpretation: alterations in growth trajectories, though not significantly affecting major folding architecture, may modulate local folding characteristics within affected regions and their neighbors. Furthermore, by disentangling the relative contributions of geometry and growth models, we found that primary folding patterns are more strongly governed by geometrical factors, including the initial cortical surface configuration and cortical thickness. In contrast, growth functions act more as dynamic modulators, influencing the magnitude and distribution of tissue deformation through their growth ratios.

While the present framework successfully captures key aspects of cortical folding, several limitations also point toward opportunities for refinement. First, axonal fiber growth was not explicitly represented; its potential contribution was approximated through the imposed spatial growth heterogeneity. Yet, developmental processes such as axonal elongation^65^, pathfinding^66^, and fiber organization^67^ have been implicated in the emergence of cortical folds^50^. Second, white matter was modeled as a homogeneous material with isotropic growth, without accounting for its internal anatomical structures. Incorporating these structural details in future models may improve the accuracy of folding predictions^5^. Additionally, spatial variations in mechanical properties were not considered, despite their recognized influence on brain biomechanics^68^. Nonetheless, the framework is inherently extensible and can readily integrate such regional heterogeneities in growth and material composition, paving the way for increasingly comprehensive and biologically realistic models of cortical development.

Collectively, this work establishes an imaging-informed, whole-brain modeling framework that mechanistically links regional growth heterogeneity to cortical folding. By uniting population MRI with biophysical modeling, the framework offers a quantitative bridge between developmental imaging and physical theory to capture the spatiotemporal complexity of cortical morphogenesis. Beyond reproducing anatomically faithful folding patterns, it provides a quantitative and extensible platform for probing the developmental origins of neurodevelopmental disorders and for generating high-fidelity synthetic datasets that support data-driven neuroscience.

## Methods

### Brain imaging data

To characterize regional growth models, we assembled longitudinal measurements of brain surface area and cortical thickness from the developing Human Connectome Project (dHCP)^69^, which is a publicly available resource focused on early brain development. To ensure reconstruction and registration fidelity, we selected 120 scans (60 males, 60 females) aged 22–40 GWs to form the finalized study dataset. Sex differences were not considered given the broad developmental focus of this study. All images were processed with the infant dedicated computational pipeline, iBEAT V2^70^, to reconstruct high-quality cortical surfaces, followed by cross-subject surface registration in spherical space to ensure vertex-wise anatomical correspondence. For surface partitioning, we applied our previously developed developmental brain atlas^4^, which divides the cortex into 36 regions (18 per hemisphere). The parcellation was constructed using a data-driven method, non-negative matrix factorization (NMF), that groups cortical vertices into regions based on shared developmental patterns of surface area expansion^4^. Regional measurements of cortical surface area and thickness across gestational age were then extracted. These longitudinal profiles served as the basis for further characterization of regional growth models.

### Symbolic regression for growth models characterization

We used symbolic regression to characterize regional growth models. Inspired by Darwinian principles of natural selection, symbolic regression automatedly discovers mathematical relationships directly from data without assuming a predefined functional form, thereby offering high flexibility in model discovery. Symbolic regression is implemented through genetic programming, where candidate functions are efficiently represented as binary tree structures composed of variables, mathematical operators (unary or binary), and constants (Fig. S2a). During training, the algorithm emulates evolutionary processes using tree-based operations, such as evaluation, selection, mutation, and crossover. Mutation introduces diversity by randomly modifying nodes within an expression tree, while crossover generates new candidate functions by recombining components from different parent trees. These processes are repeated iteratively until either an optimal function is identified, or the maximum number of generations is reached.

To depict surface area expansion and cortical thickness variation, we decomposed cortical growth into two independent modes: tangential (in-plane) growth and radial (out-of-plane) growth. For computational implementation, the data were converted into unitless growth ratios and a virtual time variable using the following formulas:

#(1) $$g_{t}(t)=\sqrt{\backslash \text{raisebox}1ex\$S(t)\$/\backslash \text{raisebox}-1ex\$S_{0}\$},g_{r}(t)=\backslash \text{raisebox}1ex\$T(t)\$/\backslash \text{raisebox}-1ex\$T_{0}\$,\;t=\frac{GA(t)-GA_{0}}{GA_{max}-GA_{0}}$$

where $g_{t}(t)$ and $g_{r}(t)$ denote tangential and radial growth ratio, respectively; $S_{0}$ and $T_{0}$ are the initial surface area and cortical thickness measured at 22 GWs ($GA_{0}$); and $GA_{\text{max}}$ corresponds to the maximum gestational age in the dataset (40 GWs). After conversion, t∈(0,1) serves as the single input variable for the symbolic regression algorithm to identify the optimal growth function g(t) for each region (Fig. S2b).

All symbolic regression analyses were performed using PYSR^71^, an open-source package built on Julia. Training was restricted to a limited operator set: binary operators included addition (+), multiplication (*), and polynomial terms ($t^{n}$), while unary operators were constrained to exponential (exp), hyperbolic tangent (tanh), natural logarithmic (ln) functions. Each training run was capped at 30 minutes or 1,000 iterations, whichever occurred first, as most qualified models converged within this range during preliminary testing. The maximum expression tree depth was set to 10, and model complexity was constrained to 100. We used the default convergence criterion (“best”) to guide the model selection process, which efficiently balances fitting accuracy and model simplicity. To satisfy growth ratio normalization, the predicted growth function was constrained to g(t=0)≡1, implemented through a penalty term in the customized objective function. For each brain region, training was repeated at least three times, and the best-performing candidate was selected as the final model. All analyses were conducted on a Legion PC equipped with a 12-core Intel Core i9–14900HX 2.2GHz CPU and 32GB of RAM.

### Biophysics in modeling brain folding

Cortical folding arises from complex biological processes, but from a mechanical perspective it can be approximated as a buckling and post-buckling problem. To capture growth-driven folding in the developing brain, we adopt the well-established differential growth theory, which attributes folding to deformation mismatch between the rapidly expanding cortical layer and the more slowly growing white matter^72^. The deformation kinematics can be described within the framework of continuum mechanics. Firstly, we consider a one-to-one mapping between the undeformed and deformed continuum body, denoted as \varvecx=ϕ(\varvecX,t), which carries a material particle \varvecX in the reference configuration ${ℬ}_{0}$ to its position \varvecx in the current configuration ${ℬ}_{t}$. This transformation is characterized by the deformation gradient, $\backslash varvecF=\nabla_{x}\phi (\backslash varvecX,t)$. Following the principle of multiplicative decomposition, the deformation process can be interpreted as a combination of stress-free growth and elastic accommodation. Accordingly, both the deformation gradient \varvecF and Jacobian determinant J, which quantifies volumetric change, are decomposed into elastic and growth components:

#(2) $$F=\backslash varvecF^{e}•\backslash varvecF^{g},\;J=det\backslash varvecF=J^{e}•J^{g}\cdot$$

For the growth component $\backslash varvecF^{g}$, we modeled gray matter growth as orthotropic, comprising both the in-plane surface expansion and out-of-plane thickness variation:

#(3) $$\backslash varvecF^{g}=g_{t}I+\left(g_{r}-g_{t}\right)\backslash \hat{varvec}n⊗\backslash \hat{varvec}n,\;J^{g}=det\backslash varvecF^{g},$$

where $g_{t}$ and $g_{r}$ are the tangential (in-plane) and radial (out-of-plane) growth ratios, respectively, with their expressions $g_{t}=g_{t}(t)$ and $g_{r}=g_{r}(t)$ derived from symbolic regression. For white matter, we assume isotropic growth $\left(g_{t}=g_{r}\right)$, implying uniform expansion in all directions, a simplification widely supported in the literature^10,23,25^. The vector $\backslash \hat{\text{varvec}n}$ denotes the local surface normal, oriented outward in the radial direction.

For the elastic component $\backslash varvecF^{e}$, we followed previous brain-folding models and adopted a standard neo-Hookean hyperelastic model to describe the constitutive behavior of both gray and white matters^19^. The strain energy density function is expressed in terms of the elastic deformation gradient $\backslash varvecF^{e}$ and its Jacobian $J^{e}$,

#(4) $$W\left(\backslash varvecF^{e}\right)=\frac{1}{2}K{ln}^{2}\left(J^{e}\right)+\frac{1}{2}\mu \left(\backslash varvecF^{e}:\backslash varvecF^{e}-2lnJ^{e}-3\right),$$

where K and μ are bulk and shear moduli, respectively. From thermodynamic principles, the first Piola-Kirchhoff stress \varvecP is work-conjugate to the deformation gradient \varvecF, and specially related to $\backslash varvecF^{e}$:

#(5) $$P=\frac{\partial W}{\partial \backslash varvecF}=\frac{\partial W\left(\backslash varvecF^{e}\right)}{\partial \backslash varvecF^{e}}:\frac{\partial \backslash varvecF^{e}}{\partial \backslash varvecF}=\backslash varvecP^{e}•\backslash varvecF^{g-T}.$$

The Piola stress enters the standard balance of linear momentum, which governs mechanical equilibrium. In the absence of body forces, this reduces to the vanishing divergence of the first Piola-Kirchhoff stress:

#(6) DivP=0.

In summary, gray and white matter growth defines a stress-free configuration, which must be balanced by the elastic deformation arising from material confinement. The neo-Hookean model provides this elastic response, ensuring mechanical compatibility and preventing unphysical deformations.

### Computational model of the developing brain

To realistically model cortical folding in the prenatal brains, we used reconstructed cortical surfaces at 22 GWs as the initial simulation geometry. As shown in Fig. S4a, the gray and white matter surfaces were registered and parcellated according to the developmental atlas. Solid models were reconstructed, merged, and meshed to produce a high-quality hexahedral volume. Hexahedral meshes were chosen over tetrahedral meshes for their superior robustness and efficiency in large-scale brain folding simulations. A mesh size of 0.4 mm was used, resulting in ~ 1,600,000 hexahedral elements for a whole-brain model. As seen in Fig. S4b, interior elements formed uniform voxels, while boundary elements were split, reprojected, and smoothed to preserve surface conformity. Elements on both sides of the gray–white interface shared faces and nodes, ensuring geometric and mechanical continuity. This meshing strategy ensured at least five elements across cortical thickness, providing sufficient resolution to capture folding development in finite element simulation^23^. Mesh quality was evaluated using scaled Jacobian and edge ratio metrics, with most elements close to 1, confirming that the mesh satisfied the requirements for reliable simulation (Fig. S4c). After meshing, gray matter elements were subdivided into 19 regions according to cortical surface parcellation for implementing regional growth in the modeling (Fig. S4d). To maintain consistency with anatomical parcellation, the regional convoluted surface was constructed from gray and white matter surfaces sharing the same regional index, and enclosed elements were assigned to the corresponding region. Elements near regional interfaces were allocated to the adjacent region with the higher index. Furthermore, orthotropic growth was enforced by assigning each gray matter element a material orientation, calculated as the normal vector from the element centroid to the nearest vertex on the cortical surface (Fig. S4e).

Brain folding was simulated as thermal expansion in ABAQUS (Dassault Systems, Paris, France) with the dynamic-explicit solver, considering the analogy between the volumetric growth and thermal expansion^73^. Both gray and white matter were modeled as nearly incompressible neo-Hookean materials, with comparable shear stiffness values of 0.65 kPa and 0.68 kPa, respectively^74^. Regional growth models $g_{r}\;(t)$ and $g_{t}\;(t)$, derived from symbolic regression, were applied to the gray matter through a user-defined subroutine *VUEXPAN.* Following previous studies^19^, we applied a sigmoid shape growth profile across cortical depth to reflect the layered growth patterns observed in cortical development^6,7^ (Fig. S4f). In contrast, the white matter was modeled using an isotropic growth model that captures its volumetric changes during normal brain development (Fig. S6). Free boundary conditions were imposed on the cortical surface, together with a self-contact constraint to prevent self-penetration. A uniform pressure was applied to represent the mechanical confinement of the meninges and developing skull^23^. The total simulation time was set to the maximal rescale gestational time (t=1). To avoid computational instabilities, the maximum time step was set as $\Delta t=0.05a\sqrt{\rho /K}$, where a is the average mesh size, ρ is the mass density, K is bulk modulus^25^. Detailed mechanical parameters are provided in Table. S2. All simulations were performed on a Dell Alienware desktop equipped with an Intel^®^ Core^™^ Ultra 9 285K processor (24 cores) @ 3.7 GHz, and 64 GB of RAM. For a single subject, the simulation of brain development from 22 to 40 GWs required approximately 12 hours of computation.

### Postprocessing analyses and quantitative metrics

In total, we simulated folding development for 10 brains (2 hemispheres per case) at 22 GWs. Simulation output files were exported and analyzed with in-house postprocessing codes. At selected simulation stages, node coordinates were extracted to reconstruct the cortical surfaces. For regional analysis, alignment between simulated and real brain parcellations was achieved by matching vertex positions between the undeformed simulated surface and the real brain geometry at 22 GWs. The assigned regional indices were directly propagated to subsequent deformed states, as the simulation preserved the original cortical surface connectivity and maintained geometry without distortion. Notably, the framework allows cortical surfaces to be reconstructed at any simulation moment, allowing continuous tracking of folding progression that cannot be fully captured by longitudinal imaging. From the reconstructed cortical surfaces, multiple quantitative metrics were calculated to evaluate the accuracy of folding simulations. These include global and local gyrification index, major sulcus count, sulcal depth, curvature measures, enabling systematic comparisons at whole-brain, regional, and vertex levels.

#### Global gyrification index:

To quantify the folding complexity of the brain surface, we employed the global gyrification index (GI), a widely used whole-brain metric. The global GI is defined as the ratio between the total cortical surface area and the area of its enclosing convex hull^19^. In our implementation, the convex hull was approximated using an alpha-shape surface, which fully encloses the cortex while minimizing the enclosing area to provide a more accurate estimate.

### Local gyrification index

In addition to the global GI, we quantified regional folding complexity using the local gyrification index. The local GI complements the global measure by capturing spatial variations in cortical folding and providing a region- or vertex-wise characterization. To enhance accuracy, we applied the anisotropic kernel method^75^, which uses wavefront propagation to construct spatially adaptive kernels that conform to cortical geometry, elongating along gyral crowns and sulcal fundi while remaining uniform over flatter regions. Compared with conventional kernels, this approach provides more reproducible local gyrification estimates that better reflect biologically relevant variations. Herein, the local GI values were computed at each vertex of the cortical surface.

### Sulcal depth

Sulcal depth is another widely used quantitative descriptor of cortical folding. Several approaches have been proposed for computing sulcal depth, differing in their choice of reference surface and distance metric. In this study, we defined sulcal depth as the Euclidean distance between the cortical surface and its convex hull, consistent with the definition used for calculating the global GI^27^. For each vertex on the cortical mesh, the nearest projection onto the triangular facets of the convex hull was identified, and the minimal Euclidean distance was taken as the sulcal depth at that vertex. Given the convexity of the hull, the shortest distance is uniquely defined, ensuring stable vertex-wise measurements across the surface.

### Curvatures

We consider the Weingarten curvature matrix to quantify curvatures in all directions. Its eigenvalues define the principal curvatures, $k_{1}$ and $k_{2}$, corresponding to the maximum and minimum normal curvatures. From these, the mean curvature is given by $k_{H}=\left(k_{1}+k_{2}\right)/2$, and the Gaussian curvature by $k_{G}=k_{1}•k_{2}$. Among these descriptors, mean curvature has been widely used in cortical folding studies. However, its magnitude depends on surface size and geometry, making it sensitive to brain scale. To obtain a scale-independent measure, we adopted a dimensionless mean curvature^27^, defined by multiplying $k_{H}$ with a characteristic length $l_{c}$

#(7) $$l_{c}=\sqrt{A_{s}/4\pi }\;\Rightarrow \;k_{H}^{*}=k_{H}•l_{c},$$

where $A_{s}$ is the surface area. Dimensionless mean curvatures were computed at each vertex of the triangular mesh and, for simplicity, are hereafter denoted as “mean curvature”. In addition, we defined the shape index (SI) as another scale-independent descriptor:

#(8) $$SI=\frac{2}{\pi }arctan\left(\frac{k_{H}}{\sqrt{k_{H}^{2}-k_{G}}}\right),$$

with values confined to [−1, 1]. Unlike raw curvature values, this normalized metric provides a normalized characterization of local surface types. Specifically, negative values correspond to concave features such as sulci, positive values to convex features such as gyri, and values near zero to saddle-like transitional regions^29^.

### Sulcus number estimation

To quantify major sulci, we employed a curvature- and size-based filtering approach. Sulcal regions were identified as contiguous patches of the cortical surface with mean curvature value less than − 0.05. To ensure robustness and to exclude spurious indentations, we further required a minimal surface area of 10 mm^2^ and at least 60 vertices per patch. We note that this threshold-based method may underestimate the number of minor or tertiary sulci, which can be functionally relevant but are easily smoothed out by curvature filtering or mesh resolution. While curvature and size thresholds are empirically defined, this method offers a straightforward means of quantifying gross sulcus counts and supporting consistent comparison between real and simulated brains. Similar curvature-based criteria have been applied in surface-based morphometry studies of cortical folding^76^.

#### Spherical mapping, sulcal landmarks, and similarity assessment:

To facilitate comparison of cortical folding patterns, we projected the brain morphology and associated quantitative measures onto a unit sphere. This was achieved using the FLASH algorithm for spherical mapping^77^. Surface registration is a necessary step to ensure meaningful cross-subject comparison. Since the real brain morphology had already been registered during cortical surface mapping, it was sufficient to build a mapping between the reconstructed brain and the undeformed simulated brain. This mapping, propagated across the computational steps, was obtained through one-to-one distance-based vertex matching once the computational mesh was constructed. For consistency, we defined two element intersections as the north and south poles, respectively: the intersection of regions 6, 15, and 16 as the north pole, and the intersection of regions 9, 17, and 18 as the south pole (Fig. 3c).

The sulcal landmarks (Fig. 3) were manually extracted and visualized using ParaView. We focused on six major sulci, including central sulcus, postcentral sulcus, intraparietal sulcus, superior temporal sulcus, lateral sulcus, and inferior frontal sulcus, which were selected for their anatomical prominence and accessibility of extraction. These sulci also carry important functional relevance, as they delineate boundaries of primary sensorimotor cortices (central and postcentral sulcus), visuospatial and attentional networks (intraparietal sulcus), auditory and language-related regions (superior temporal and lateral sulcus), and higher-order executive areas (inferior frontal sulcus). Although the alignment is not restricted to these six sulci, a more detailed landmark alignment analysis lies beyond the scope of this study. Here, we highlight these six representative sulci to demonstrate the capability of our whole-brain framework to model brain morphology with anatomical fidelity.

After surface registration, the similarity s between distributions of a quantitative metric d in real brains ($S_{\mathrm{real}}$) and simulated brains ($S_{\mathrm{simu}}$) was evaluated using the following formula^39^:

#(9) $$s\left(S_{real},S_{simu}\right)=1-\frac{1}{2\sqrt{N}}{\left\|d_{real}\circ f_{rs}-d_{simu}\right\|}_{2},$$

where N is the total number of vertices; $f_{rs}$ is the mapping from the real brain to the simulated brain, accommodating the higher vertex density of the real mesh; $\|•{\|}_{2}$ denotes Euclidean norm, i.e., ${\left\|d_{\mathrm{real}}\circ f_{rs}-d_{\mathrm{simu}}\right\|}_{2}={\left(\sum_{i=1}^{N}\;{\left(d_{\mathrm{real}}\circ f_{rs}-d_{\mathrm{simu}}\right)}^{2}\right)}^{1/2}$. Noted, to ensure that the similarity measure always falls within the range of [0,1], the quantitative metric d must be normalized to [−1,1], with a higher value of indicating greater similarity^39^. In this study, we calculate the similarity based on the distribution of the shape index, which inherently lies within the required range. By contrast, the similarity score used in Fig. 5c was defined as $exp\left(-\left|log\left(d_{\mathrm{simu}}/d_{\mathrm{real}}\right)\right|\right)$, where $d_{\mathrm{simu}}$ and $d_{\mathrm{real}}$ denotes the metric measure of simulated and real brain cases, respectively. This similarity score has values between 0 and 1, with 1 indicating perfect agreement. The relative percentage errors in Fig. 7 was defined as $\left(d_{stu}-d_{\mathrm{ref}}\right)/d_{\mathrm{ref}}*100\%$, where $d_{stu}$ and $d_{\mathrm{ref}}$ denotes the metric measure of studied and reference brain case, respectively.

### Growth profile definition

To investigate the influence of growth trajectories on cortical folding simulation, we introduced three additional growth models based on a high-order polynomial (POL), hyperbolic tangent (HYT), and Gompertz distribution (GOM), respectively (Fig. 7a). These functional forms have been used in modeling tissue growth processes such as tumor progression^78^ and the development of myelinated white matter in the human brain^79^. The growth functions are defined as

#(10) $$g_{POL}(t)=c_{1}*t^{c_{2}},$$

#(11) $$g_{HYT}(t)=c_{3}*tanh\left(c_{4}*t\right)+1,$$

#(12) $$g_{GOM}(t)=c_{5}*exp\left(-exp\left(-c_{6}*\left(t-c_{7}\right)\right)\right)+1,$$

where $c_{i}$ are constants. As an illustrative case, we considered the tangential growth model of region 1 (Fig. 7a), for which the symbolic regression (SYR) predicted model is given by $g_{SYR}(t)=1.46*t+1$. Accordingly, the parameter values were set as: POL, $c_{1}=1.46,\;c_{2}=3$, HYT, $c_{3}=1.46*1.012,\;c_{4}=2.6$, and GOM, $c_{5}=1.46*1.038,\;c_{6}=6,\;c_{7}=0.45$. The parameter values were empirically assigned to ensure that all models converge to a consistent growth ratio at the final stage, thereby eliminating differences in absolute growth magnitude and allowing the analysis to focus exclusively on the effect of growth trajectories.

For the growth conditions shown in Fig. 5, the isotropic growth with a uniform profile (IGU) case was defined by linear growth functions $g_{r}(t)=g_{t}(t)=\sqrt{8}*t$, whereas the tangential growth with a uniform profile (TGU) case used $g_{r(t)}=1,\;g_{t}(t)=\sqrt{8}*t$ The chosen growth ratio values follow those commonly adopted in previous studies for modeling simplification^10,25^.

### Statistical analysis

Statistical analyses were performed using non-parametric tests given the small sample size (n = 10 per group), although data satisfied normality as assessed by the Shapiro–Wilk test. In Fig. 5d, difference in the sulcus counts among five growth conditions (Real, OGR, TGR, TGU, and IGU; n = 50, 10 per group) were first evaluated using the Kruskal–Wallis test to evaluate the null hypothesis that group medians were equal. When the overall test was significant (*P* < 0.05), post hoc pairwise comparisons were carried out using Dunn’s test with Bonferroni correction for multiple testing. In Fig. S5e, paired comparisons of sulcal counts between left and right hemispheres (n = 20, 10 per hemisphere) were assessed using the Wilcoxon signed-rank test. The null hypothesis was that the median differences between pairs was zero. All tests were two-tailed, with P < 0.05 considered statistically significant. Statistical analyses were performed using OriginPro 2025.

## Supplementary Material

Supplementary Files

This is a list of supplementary files associated with this preprint. Click to download.

• Movie3.mp4

• Movie6.mp4

• Movie7.mp4

• Movie5.mp4

• Movie4.mp4

• Movie1.mp4

• Movie2.mp4

• Supplementaryinformation.docx

## Data Availability

The original contributions presented in the study are included in the article/supplemental material. Further inquiries can be directed to the corresponding authors. The dHCP dataset is publicly available at the Developing Human Connectome Project repository.

## References

[1] Mihailov A (2025) Burst of gyrification in the human brain after birth. Commun Biology 8:805 10.1038/s42003-025-08155-z PMC12106832 40419689

[2] Garcia KE (2018) Dynamic patterns of cortical expansion during folding of the preterm human brain. Proceedings of the National Academy of Sciences 115, 3156–3161 10.1073/pnas.1715451115 PMC5866555 29507201

[3] Wang F (2019) Developmental topography of cortical thickness during infancy. Proceedings of the National Academy of Sciences 116, 15855–15860 10.1073/pnas.1821523116 PMC6689940 31332010

[4] Huang Y (2022) Mapping developmental regionalization and patterns of cortical surface area from 29 post-menstrual weeks to 2 years of age. Proceedings of the National Academy of Sciences 119, e2121748119 10.1073/pnas.2121748119 PMC9388141 35939665

[5] Sun T, Hevner RF (2014) Growth and folding of the mammalian cerebral cortex: from molecules to malformations. Nat Rev Neurosci 15:217–232 24646670 10.1038/nrn3707 PMC4107216

[6] Ball G (2024) Molecular signatures of cortical expansion in the human foetal brain. Nat Commun 15:9685 39516464 10.1038/s41467-024-54034-2 PMC11549424

[7] Qian X (2025) Spatial transcriptomics reveals human cortical layer and area specification. Nature10.1038/s41586-025-09010-1 PMC12328223 40369074

[8] Schmitt JE, Raznahan A, Liu S, Neale MC (2021) The Heritability of Cortical Folding: Evidence from the Human Connectome Project. Cereb Cortex 31:702–715 32959043 10.1093/cercor/bhaa254 PMC7727360

[9] Voorhies WI, Miller JA, Yao JK, Bunge SA, Weiner KS (2021) Cognitive insights from tertiary sulci in prefrontal cortex. Nat Commun 12:5122 34433806 10.1038/s41467-021-25162-w PMC8387420

[10] Tallinen T (2016) On the growth and form of cortical convolutions. Nat Phys 12:588–593

[11] Hazlett HC (2017) Early brain development in infants at high risk for autism spectrum disorder. Nature 542:348–351 28202961 10.1038/nature21369 PMC5336143

[12] Jaffe AE (2018) Developmental and genetic regulation of the human cortex transcriptome illuminate schizophrenia pathogenesis. Nat Neurosci 21:1117–1125 30050107 10.1038/s41593-018-0197-y PMC6438700

[13] Oegema R (2020) International consensus recommendations on the diagnostic work-up for malformations of cortical development. Nat Reviews Neurol 16:618–635 10.1038/s41582-020-0395-6 PMC7790753 32895508

[14] Van Essen DC (1997) A tension-based theory of morphogenesis and compact wiring in the central nervous system. Nature 385:313–318 9002514 10.1038/385313a0

[15] Nie J (2011) Axonal Fiber Terminations Concentrate on Gyri. Cereb Cortex 22:2831–2839 22190432 10.1093/cercor/bhr361 PMC3491768

[16] Chavoshnejad P (2021) Role of axonal fibers in the cortical folding patterns: A tale of variability and regularity. Brain Multiphysics 2:100029

[17] Garcia KE, Wang X, Kroenke CD (2021) A model of tension-induced fiber growth predicts white matter organization during brain folding. Nat Commun 12:6681 34795256 10.1038/s41467-021-26971-9 PMC8602459

[18] Zarzor MS, Kaessmair S, Steinmann P, Blümcke I, Budday S (2021) A two-field computational model couples cellular brain development with cortical folding. Brain Multiphysics 2

[19] Tallinen T, Chung JY, Biggins JS, Mahadevan L (2014) Gyrification from constrained cortical expansion. Proceedings of the National Academy of Sciences 111, 12667–12672 10.1073/pnas.1406015111 PMC4156754 25136099

[20] Razavi MJ, Zhang T, Li X, Liu T, Wang X (2015) Role of mechanical factors in cortical folding development. Phys Rev E 92:032701 10.1103/PhysRevE.92.032701 26465492

[21] Wang X (2019) in. 41st Annual International Conference of the IEEE Engineering in Medicine and Biology Society (EMBC). 146–149

[22] Alenyà M (2022) Computational pipeline for the generation and validation of patient-specific mechanical models of brain development. Brain Multiphysics 3

[23] Solhtalab A, Guo Y, Gholipour A, Dai W, Razavi MJ (2025) Mechanics of the Spatiotemporal Evolution of Sulcal Pits in the Folding Brain. Hum Brain Mapp 46:e70332 40862306 10.1002/hbm.70332 PMC12381649

[24] Budday S, Steinmann P, Goriely A, Kuhl E (2015) Size and curvature regulate pattern selection in the mammalian brain. Extreme Mech Lett 4:193–198

[25] Wang X (2021) The influence of biophysical parameters in a biomechanical model of cortical folding patterns. Sci Rep 11:7686 33833302 10.1038/s41598-021-87124-y PMC8032759

[26] Jalil Razavi M, Zhang T, Liu T, Wang X (2015) Cortical folding pattern and its consistency induced by biological growth. Sci Rep 5:14477 26404042 10.1038/srep14477 PMC4585925

[27] Balouchzadeh R, Bayly PV, Garcia KE (2023) Effects of stress-dependent growth on evolution of sulcal direction and curvature in models of cortical folding. Brain Multiphysics 4:100065 38948884 10.1016/j.brain.2023.100065 PMC11213281

[28] Makropoulos A (2016) Regional growth and atlasing of the developing human brain. NeuroImage 125:456–478 26499811 10.1016/j.neuroimage.2015.10.047 PMC4692521

[29] Demirci N, Holland MA (2024) Scaling patterns of cortical folding and thickness in early human brain development in comparison with primates. Cereb Cortex 34:bhad462 38271274 10.1093/cercor/bhad462

[30] Xu X (2022) Spatiotemporal Atlas of the Fetal Brain Depicts Cortical Developmental Gradient. J Neurosci 42:9435 36323525 10.1523/JNEUROSCI.1285-22.2022 PMC9794379

[31] Liang X (2024) Structural connectome architecture shapes the maturation of cortical morphology from childhood to adolescence. Nat Commun 15:784 38278807 10.1038/s41467-024-44863-6 PMC10817914

[32] Hill J (2010) Similar patterns of cortical expansion during human development and evolution. Proceedings of the National Academy of Sciences 107, 13135–13140 10.1073/pnas.1001229107 PMC2919958 20624964

[33] Namburete AIL (2023) Normative spatiotemporal fetal brain maturation with satisfactory development at 2 years. Nature 623:106–114 37880365 10.1038/s41586-023-06630-3 PMC10620088

[34] Natu VS (2021) Infants’ cortex undergoes microstructural growth coupled with myelination during development. Commun Biology 4:1191 10.1038/s42003-021-02706-w PMC8516989 34650227

[35] Yun HJ (2022) Quantification of sulcal emergence timing and its variability in early fetal life: Hemispheric asymmetry and sex difference. NeuroImage 263:119629 36115591 10.1016/j.neuroimage.2022.119629 PMC10011016

[36] Jensen MA (2023) A motor association area in the depths of the central sulcus. Nat Neurosci 26:1165–1169 37202552 10.1038/s41593-023-01346-z PMC10322697

[37] Norman-Haignere SV (2025) Temporal integration in human auditory cortex is predominantly yoked to absolute time. Nat Neurosci10.1038/s41593-025-02060-8 PMC12586173 40968242

[38] Demirci N, Holland MA (2022) Cortical thickness systematically varies with curvature and depth in healthy human brains. Hum Brain Mapp 43:2064–2084 35098606 10.1002/hbm.25776 PMC8933257

[39] Yin S (2025) Morphogenesis and morphometry of brain folding patterns across species. eLife 10.7554/eLife.107138 PMC12747518 41459643

[40] Costa Campos Ld, Hornung R, Gompper G, Elgeti J, Caspers S (2021) The role of thickness inhomogeneities in hierarchical cortical folding. NeuroImage 231:117779 33548459 10.1016/j.neuroimage.2021.117779

[41] Ben Amar M (2025) Wrinkles, creases, and cusps in growing soft matter. Rev Mod Phys 97:015004

[42] Van Essen DC (2019) Cerebral cortical folding, parcellation, and connectivity in humans, nonhuman primates, and mice. Proceedings of the National Academy of Sciences 116, 26173–26180 10.1073/pnas.1902299116 PMC6936571 31871175

[43] Di Donato N (2017) Lissencephaly: Expanded imaging and clinical classification. Am J Med Genet A 173:1473–1488 28440899 10.1002/ajmg.a.38245 PMC5526446

[44] Golden JA, Harding BN (2010) Unfolding polymicrogyria. Nat Reviews Neurol 6:471–472 10.1038/nrneurol.2010.118 20811463

[45] Edlow BL (2019) 7 Tesla MRI of the ex vivo human brain at 100 micron resolution. Sci Data 6:244 31666530 10.1038/s41597-019-0254-8 PMC6821740

[46] Vezain M (2018) A de novo variant in ADGRL2 suggests a novel mechanism underlying the previously undescribed association of extreme microcephaly with severely reduced sulcation and rhombencephalosynapsis. Acta Neuropathol Commun 6:109 30340542 10.1186/s40478-018-0610-5 PMC6195752

[47] Park SH (2013) A Forensic Autopsy Case of Lissencephaly for Evaluating the Possibility of Child Abuse. Korean J Lab Med 37:84–89

[48] Coman D (2017) X-Linked Lissencephaly With Absent Corpus Callosum and Abnormal Genitalia: An Evolving Multisystem Syndrome With Severe Congenital Intestinal Diarrhea Disease. Child Neurol Open 4:2329048× 29152528 10.1177/2329048X17738625 PMC5680935

[49] Atwood S (2024) A Rare Case of Polymicrogyria in an Elderly Individual. Saint Louis University 10.7759/cureus.74300 PMC11665267 39717325

[50] Garcia KE, Kroenke CD, Bayly PV (2025) Mechanical stress connects cortical folding to fiber organization in the developing brain. Trends Neurosci 48:395–402 40307105 10.1016/j.tins.2025.04.001 PMC12439404

[51] Kroenke CD, Bayly PV (2018) How Forces Fold the Cerebral Cortex. J Neurosci 38:767 29367287 10.1523/JNEUROSCI.1105-17.2017 PMC5783962

[52] Nowakowski TJ (2017) Spatiotemporal gene expression trajectories reveal developmental hierarchies of the human cortex. Science 358:1318–1323 29217575 10.1126/science.aap8809 PMC5991609

[53] Pollen AA (2015) Molecular identity of human outer radial glia during cortical development. Cell 163:55–67 26406371 10.1016/j.cell.2015.09.004 PMC4583716

[54] Akula SK, Exposito-Alonso D, Walsh CA (2023) Shaping the brain: The emergence of cortical structure and folding. Dev Cell 58:2836–2849 38113850 10.1016/j.devcel.2023.11.004 PMC10793202

[55] Budday S, Steinmann P (2018) On the influence of inhomogeneous stiffness and growth on mechanical instabilities in the developing brain. Int J Solids Struct 132:31–41

[56] Chavoshnejad P (2023) An integrated finite element method and machine learning algorithm for brain morphology prediction. Cereb Cortex 33:9354–9366 37288479 10.1093/cercor/bhad208 PMC10393506

[57] Yuan X (2025) Flexible Individualized Developmental Prediction of Infant Cortical Surface Maps via Intensive Triplet Autoencoder. IEEE Trans Med Imaging 44:3110–3122 40257887 10.1109/TMI.2025.3562003 PMC12765240

[58] Lin Z (2025) A Physics-Informed Neural Network Framework for Simulating Creep Buckling in Growing Viscoelastic Biological Tissues. arXiv preprint arXiv:2506.18565

[59] Zhang L (2025) Learning lifespan brain anatomical correspondence via cortical developmental continuity transfer. Med Image Anal 99:103328 39243599 10.1016/j.media.2024.103328 PMC11609030

[60] Zhao Y, Xu Z (2024) A data-driven approach to morphogenesis under structural instability. Cell Rep Phys Sci 5

[61] Cauwenberghs G (2013) Reverse engineering the cognitive brain. Proceedings of the National Academy of Sciences 110, 15512–15513 10.1073/pnas.1313114110 PMC3785732 24029019

[62] de Jager EJ, Risser L, Mescam M, Fonta C, Beaudet A (2022) Sulci 3D mapping from human cranial endocasts: A powerful tool to study hominin brain evolution. Hum Brain Mapp 43:4433–4443 35661328 10.1002/hbm.25964 PMC9435008

[63] Kochiyama T (2018) Reconstructing the Neanderthal brain using computational anatomy. Sci Rep 8:6296 29700382 10.1038/s41598-018-24331-0 PMC5919901

[64] Nickl-Jockschat T (2012) Brain structure anomalies in autism spectrum disorder–a meta-analysis of VBM studies using anatomic likelihood estimation. Hum Brain Mapp 33:1470–1489 21692142 10.1002/hbm.21299 PMC4801488

[65] Van Essen DC (2020) A 2020 view of tension-based cortical morphogenesis. Proceedings of the National Academy of Sciences 117, 32868–32879 10.1073/pnas.2016830117 PMC7780001 33323481

[66] Solhtalab A, Foroughi AH, Pierotich L, Razavi MJ (2025) Stress landscape of folding brain serves as a map for axonal pathfinding. Nat Commun 16:1187 39885152 10.1038/s41467-025-56362-3 PMC11782574

[67] Chavoshnejad P (2023) Mechanical hierarchy in the formation and modulation of cortical folding patterns. Sci Rep 13:13177 37580340 10.1038/s41598-023-40086-9 PMC10425471

[68] Griffiths E, Hinrichsen J, Reiter N, Budday S (2023) On the importance of using region-dependent material parameters for full-scale human brain simulations. Eur J Mechanics-A/Solids, 104910

[69] Project DHC (2024) Accessed Developing Human Connectome Project. http://www.developingconnectome.org

[70] Wang L (2023) iBEAT V2.0: a multisite-applicable, deep learning-based pipeline for infant cerebral cortical surface reconstruction. Nat Protoc 18:1488–1509 36869216 10.1038/s41596-023-00806-x PMC10241227

[71] Cranmer M (2023) Interpretable machine learning for science with PySR and SymbolicRegression. jl. arXiv preprint arXiv:2305.01582

[72] Garcia KE, Kroenke CD, Bayly PV (2018) Mechanics of cortical folding: stress, growth and stability. Philosophical Trans Royal Soc B: Biol Sci 373:20170321 10.1098/rstb.2017.0321 PMC6158197 30249772

[73] Cao Y, Jiang Y, Li B, Feng X (2012) Biomechanical modeling of surface wrinkling of soft tissues with growth-dependent mechanical properties. Acta Mech Solida Sin 25:483–492

[74] Hou J (2025) Mechanical characterization of brain tissue: experimental techniques, human testing considerations, and perspectives. Acta Biomater10.1016/j.actbio.2025.07.046 PMC13428356 40706781

[75] Lyu I, Kim SH, Girault JB, Gilmore JH, Styner M (2018) A. A cortical shape-adaptive approach to local gyrification index. Med Image Anal 48:244–258 29990689 10.1016/j.media.2018.06.009 PMC6167255

[76] Zhang S (2022) Gyral peaks: Novel gyral landmarks in developing macaque brains. Hum Brain Mapp 43:4540–4555 35713202 10.1002/hbm.25971 PMC9491295

[77] Choi PT, Lam KC, Lui LM (2015) FLASH: Fast Landmark Aligned Spherical Harmonic Parameterization for Genus-0 Closed Brain Surfaces. SIAM J Imaging Sci 8:67–94

[78] Araujo RP, McElwain DL (2004) S. A linear-elastic model of anisotropic tumour growth. Eur J Appl Math 15:365–384

[79] Sadeghi N (2013) Regional characterization of longitudinal DT-MRI to study white matter maturation of the early developing brain. NeuroImage 68:236–247 23235270 10.1016/j.neuroimage.2012.11.040 PMC3693970

